# Genome Sequences of Mycobacterium smegmatis Phages Purgamenstris and PhancyPhin

**DOI:** 10.1128/MRA.01288-20

**Published:** 2021-03-04

**Authors:** Amairani D. Ramirez Rendon, Angelica Diaz-Sanchez, Ryan J. Pool, Fatin Ahmed, Aissa Mendez, Rosa C. Medellin, Fabian A. Mendoza, Richard Anderson, Marlen A. Sanchez, Jacqueline R. Ramos, Rachna Sadana, Sanghamitra Saha

**Affiliations:** a Department of Natural Sciences, University of Houston-Downtown, Houston, Texas, USA; Queens College

## Abstract

Two novel mycobacteriophages, PhancyPhin and Purgamenstris, were isolated from the Houston, Texas, area. They were isolated in the same year with the soil enrichment method using the host Mycobacterium smegmatis mc^2^ 155. They exhibit a 99.55% nucleotide identity with each other.

## ANNOUNCEMENT

Mycobacteriophages are bacterial viruses that infect host mycobacterial cells and are found in abundance in soil. With the increase in drug-resistant bacterial infections, the use of phage therapy has become an attractive and alternative tool (1). Moreover, the discovery of novel mycobacteriophages provides insights into the evolutionary history of bacterial viruses (2). As part of the Science Education Alliance Phage Hunters Advancing Genomics and Evolutionary Science (SEA-PHAGES) program (3), we report the isolation and annotation of two phages that were isolated in undergraduate biology lab courses (Biol 1101 and Biol 1102) at the University of Houston-Downtown.

PhancyPhin (coordinates 29.767149N, 95.359187W) and Purgamenstris (coordinates 29.766083N, 95.35881W) were isolated from soil samples with the soil enrichment method using the host Mycobacterium smegmatis mc^2^ 155. Soil samples were incubated in 7H9 medium at 37°C for 24 h, followed by filtration through a 0.22-μm filter. The filtrate was checked for plaque formation using a plaque assay. After purification and amplification, high-titer lysates were obtained from which genomic DNA was isolated using the Wizard DNA extraction kit (Promega). Sequencing libraries were prepared using New England Biolabs (NEB) Ultra II kits and run on an Illumina MiSeq system, yielding ∼685,000 single-end 150-base reads for PhancyPhin and 605,000 single-end 150-base reads for Purgamenstris. The raw reads were assembled using Newbler 2.9 and Consed 23 software with coverages of 615× and 1,989×, respectively, for PhancyPhin and Purgamenstris (4). Genome annotation and identification of protein-coding genes was done using DNA Master 5.23.2 (5), Glimmer 3.02 (6), GeneMark 2.5p (7), Starterator (https://seaphagesbioinformatics.helpdocsonline.com/home), Phamerator (8), BLASTp (9), and HHPRED 3.2.0 (10). Mycobacteriophages have been classified into 28 clusters; clusters are groups of phages with sequence similarity with over 50% of the genome. PhancyPhin and Purgamenstris were assigned to cluster N based on sequence analysis (2). Current data indicate that 39 sequenced phages belong to cluster N, are temperate in nature, and have well-studied viral defense systems (11).

The main features of both phages are summarized in Table 1.

**TABLE 1:** Characteristics of cluster N phages PhancyPhin and Purgamenstris

Phage name	GenBank accession no.	SRA accession no.	Genome size (bp)	Genome terminus (13-base 3′ overhang)	GC content (%)	No. of ORFs
PhancyPhin	KX756439	SRX9162540	42,454	CCCGCCGCCTTGG	66.1	69
Purgamenstris	MK524522	SRX9162541	42,595	CCCGCCGCCTTGG	66.1	70

The genome architecture of both phages is similar to that of other cluster N temperate phages, such as the presence of viral assembly and structural genes on the left arm, followed by the lysis and immunity cassette in the central region and the nonstructural genes on the right arm (1). Approximately one-third of the predicted genes in both genomes code for proteins with known functions. Both phages have siphoviridae morphology, as seen by transmission electron microscopy (TEM).

Lysin A and holin genes were found in similar positions, open reading frame 25 (ORF 25) and ORF 26, respectively, in the left arm of both phages. The immunity repressor gene (ORF 37) was found adjacent to the tyrosine integrase gene (ORF 38) in the central region of both phage genomes. No tRNAs were found in either genome. In PhancyPhin, two genes coding for HNH endonucleases were identified in the right arm, whereas in Purgamenstris, a single gene coding for HNH endonuclease was found in a similar location. These genomes exhibit a 99.55% nucleotide identity with some minor differences at the right ends of the genomes, which was determined by BLAST analysis.

### Data availability.

The GenBank and SRA accession numbers are listed in Table 1.
